# Factors Associated with Seasonal Influenza Vaccination Among Working-Age Adults in Poland: A Nationwide Cross-Sectional Survey

**DOI:** 10.3390/vaccines13090954

**Published:** 2025-09-06

**Authors:** Radosław Sierpiński, Mateusz Jankowski, Filip Raciborski

**Affiliations:** 1 Faculty of Medicine, Collegium Medicum, Cardinal Stefan Wyszynski University, 01-978 Warsaw, Poland; 2 Department of Population Health, School of Public Health, Centre of Postgraduate Medical Education, 01-813 Warsaw, Poland; 3 Department of Prevention of Environmental Hazards, Allergology and Immunology, Faculty of Health Sciences, Medical University of Warsaw, 02-091 Warsaw, Poland

**Keywords:** influenza, vaccination, flu, occupational medicine, working-age adults, workforce, economic impact, influenza-related absenteeism

## Abstract

Background: Influenza-related absenteeism causes significant economic implications. Vaccination is the most effective intervention for preventing influenza infection and its complications. This study aimed to assess the prevalence of seasonal influenza vaccination as well as to identify factors associated with seasonal influenza vaccination among working-age adults in Poland. Methods: This study is a secondary analysis of a dataset generated during the representative cross-sectional survey among adults aged 18–64 years in Poland (December 2024). In the study group (*n* = 5006), 49.9% were women. Results: Among all respondents, 16.9% declared getting vaccinated against influenza in the last 3 years: 8.2% were vaccinated several times during this period, and 8.8% were vaccinated once. There were several socio-demographic differences (*p* < 0.05) in the influenza vaccination uptake. Among working-age adults, male gender (OR: 1.83, 95% CI: 1.55–2.15, *p* < 0.001), age 18–24 years (OR: 2.63, 95% CI: 2.05–3.39; *p* < 0.001), living in cities over 100,000 residents (*p* < 0.05), having a part-time job (OR: 1.37; 95% CI: 1.08–1.73; *p* < 0.01), very good household financial situation (OR: 1.64; 95% CI: 1.19–2.24; *p* < 0.01), frequent infections throughout the year (*p* < 0.05), having chronic diseases (*p* < 0.05), taking dietary supplements regularly (OR: 1.66; 95% CI: 1.36–2.03; *p* < 0.001) and personal beliefs on doctors’ competencies (*p* < 0.05) were significantly associated with getting vaccinated against influenza in the last 3 years. Conclusions: This study revealed very low influenza vaccination coverage rates in working-age adults in Poland. Public health interventions are needed to address gaps in influenza vaccination uptake among working-age adults.

## 1. Introduction

Seasonal influenza is an acute respiratory infection caused by influenza viruses [1,2]. The disease spreads easily between people when they cough or sneeze [1]. Seasonal influenza remains a persistent global public health challenge, with around a billion cases annually, including 3–5 million cases of severe illness [3]. Vaccination is the most effective intervention for preventing influenza infection and its complications [4]. Seasonal influenza vaccination is particularly important in populations with increased risk of complications from influenza, like older adults, people with chronic diseases, people living in nursing homes, and young children [3,4]. Moreover, populations at increased risk of transmission are also at high priority for influenza vaccination [4,5].

Transmission of influenza viruses is particularly high in crowded environments and during frequent interpersonal contact [6,7]. Those conditions are common in workplace settings, especially manufacturing and commercial industries as well as educational facilities [7]. Working-age adults (18–64 years), due to numerous social interactions in work, mobility, and commuting, play an important role in the transmission of influenza viruses as vectors to vulnerable groups [7,8]. Moreover, in crowded places in the workplace, there is a high risk of transmission of influenza viruses between co-workers [7,8]. Influenza outbreaks in the workplace lead to absenteeism, when ill people stay at home, and presenteeism, when employees are working while ill [9,10,11]. Moreover, the influenza outbreaks in the workplace also cause productivity loss, increased employer healthcare costs, and operational disruptions [10,11]. Influenza-related absenteeism causes significant economic implications [10]. Due to the significant economic and organizational impact of influenza-related absenteeism, the promotion of seasonal influenza vaccination and influenza vaccination coverage are important actions to reduce the burden of influenza-related events in the workplace. Seasonal influenza vaccination is a cost-effective preventive measure that supports both employee health and workforce continuity [7,8,9,10,11].

Despite the scientific evidence on the cost-effectiveness of influenza vaccination, vaccination coverage rates in many European countries remain low [12,13]. Poland is an example of a European Union (EU) country with a low influenza vaccination coverage rate [13]. In a representative sample of adult Poles, in influenza season 2020/2021, the vaccination coverage rate was estimated at 5.5% [14]. In 2021, among older adults, the vaccination coverage rate in Poland was estimated at 10%, which was one of the lowest rates in the EU, and several times lower than in Ireland (75.4%), Denmark (75%), or the Netherlands (72.6%) [13].

In Poland, seasonal influenza vaccination is recommended to all individuals aged ≥6 months (excluding populations with medical contraindications) [14]. Influenza vaccination is fully reimbursed for children under 18 years of age, adults aged 65 years and over, and pregnant women [14]. Adults aged 18–64 are eligible for 50% reimbursement [14,15]. A person who wants to get vaccinated against influenza must obtain a prescription from a doctor, purchase the vaccine, and go to a vaccination point within the primary health care or have the vaccination done by a pharmacist at a pharmacy [14]. Employers often arrange “influenza vaccination days”, when employees can benefit from free-of-charge vaccinations in a mobile vaccination point located in the company area [7,9]. Despite the reimbursement policy and wide availability of influenza vaccination points, organizational barriers to access to influenza vaccination are still present in Poland.

Currently, there is a lack of representative data on influenza vaccination coverage rates in working-age adults. Moreover, there is a lack of data on social attitudes towards the influenza vaccination in a working-age population. Working adults are a group that can be relatively easy approached within the workplace health programs, including those on vaccination [5,7]. Moreover, influenza has a high spreading potential in workplaces, especially in industry and retail stores [7,8,9]. Detailed identification of social behaviors related to influenza vaccine uptake, as well as factors associated with attitudes towards influenza vaccination, may provide important data that can be used for health policy planning and limiting influenza epidemics in local communities, e.g., in workplaces [8,9]. These data may inform policymakers and public health authorities on potential barriers and social needs that should be addressed to increase the influenza vaccination coverage rate in Poland and reduce the burden of influenza-related absenteeism.

This study aimed to assess the prevalence of seasonal influenza vaccination as well as to identify factors associated with seasonal influenza vaccination among working-age adults in Poland.

## 2. Materials and Methods

### 2.1. Study Design and Population

Data for this secondary analysis were obtained from a dataset managed by the National Centre for Health Policy and Health Inequalities at Cardinal Stefan Wyszyński University. The dataset was collected under a contract with the Polish Ministry of Education and Science (Agreement No. MEiN/2023/DPI/2717, dated 13 October 2023) and was focused on health-related behaviors and health inequalities within the Polish population [16].

For the present study, self-reported data on seasonal influenza vaccination in the last 3 years were acquired from the complete list of survey records assessing public attitudes and behaviors toward health prevention, which were extracted from this database. The original data collection was conducted in December 2024 using a computer-assisted web interviewing (CAWI) method administered by the public opinion research agency ARC Rynek i Opinia [17]. The nationally representative sample included over 5000 adults aged 18 to 64 years. The sampling framework included stratification by gender, age, size of place of residence, and level of education. The sample design was based on demographic statistics published by Statistics Poland and was weighted to reflect the structure of the working-age adult population (18–64 years) in Poland.

Data were made available at no cost under the statutory activities of the National Centre for Health Policy and Health Inequalities, under policies on open access to publicly funded data.

The study protocol received ethical approval from the Ethics Committee at the Medical University of Warsaw (Decision No. AKBE/56/2025).

A similar approach was used in a previously published secondary data analysis based on the same data source [18].

### 2.2. Measures

Information about getting vaccinated against influenza was self-declared and was based on the following question: “Have you been vaccinated against seasonal influenza in the last 3 years?”, with three possible answers: yes, several times; yes, once; no.

Those who declared they had not been vaccinated against influenza in the last 3 years were asked an additional question about the reasons for not being vaccinated: “Why did you not get vaccinated against influenza? Please select no more than 3 most important reasons from the list below” (13 different reasons).

Moreover, questions on personal characteristics and beliefs regarding health-related issues were used. “Please indicate whether you agree or disagree with the following statements: (1) Dietary supplements are safe and effective for health; (2) Vaccinations are unnecessary if someone leads a healthy lifestyle and eats well; (3) Natural therapies, such as herbal medicine, are more effective than synthetic drugs; (4) Taking vitamin C in large amounts protects against all viral infections; (5) The vast majority of doctors are competent; (6) After receiving a diagnosis, I usually try to get a second opinion from another doctor—each statement with 5-point Likert scale. Questions on dietary supplements intake in the last 3 months, self-reported health status compared to peers, and average number of infections per year were addressed.

A detailed list of questions used in this study is presented in Supplementary File S1.

### 2.3. Data Analysis

Records obtained from the Centre for Health Policy and Health Inequalities were used to construct datasets for statistical analysis [16]. All analyses were performed using IBM SPSS Statistics software, version 29.0 (IBM Corp., Armonk, NY, USA). Demographic weighting was applied to ensure representativeness of the sample.

Descriptive statistics were presented as frequencies and proportions. The chi-squared (χ^2^) test was used to assess associations between categorical variables. A multivariable logistic regression model was developed to identify factors associated with at least one vaccination against influenza in the last 3 years (dependent variable). 11 different socio-demographic variables were considered as independent variables. The model that explain having been vaccinated against influenza at least once in the last 3 years was selected, and the model performance was evaluated using Cox and Snell R^2^ and Nagelkerke R^2^ values. Associations were expressed as odds ratios (ORs) with corresponding 95% confidence intervals (95% CI). A *p*-value of less than 0.05 was considered statistically significant.

## 3. Results

In the study group (*n* = 5006), 49.9% were women. Participants age ranged from 18 to 64 years. The mean age was 41.8 (SD = 12.59) and the median was 42. Those with higher education accounted for 30.3%, and those with secondary education accounted for 37.9%. Urban residents constituted 59.4% of the sample. 11.8% of the sample had children under 4 years of age, and 13.5% had children aged 5 to 8. The share of full-time workers was 57.2%, and another 18.0% worked part-time or casually. Among participants, 39.5% declared a good or very good financial situation, 47.1% did not suffer from any of the 13 chronic diseases analyzed, and 26.8% of the respondents assessed their health as definitely or somewhat better than that of others of the same age. Detailed data are presented in Table 1.

### 3.1. Public Attitudes Towards Seasonal Influenza Vaccination—Socio-Demographic and Health Differences

**Figure 1:**
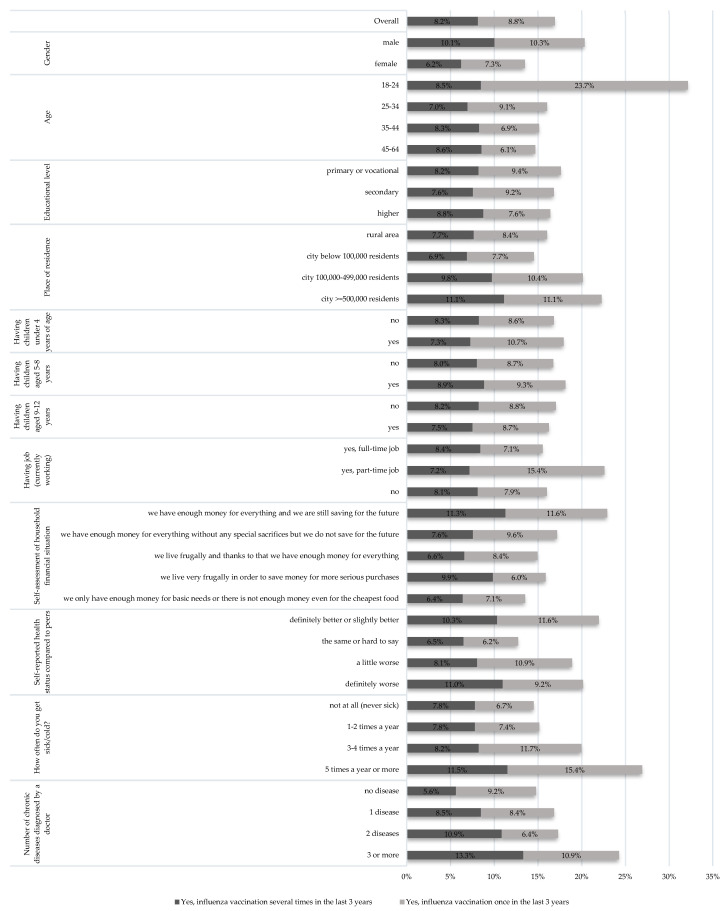
Percentage of people declaring having been vaccinated against seasonal influenza in the last 3 years, depending on socio-demographic factors and health status (*n* = 5006).

Among all respondents, 16.9% declared getting vaccinated against influenza in the last 3 years: 8.2% were vaccinated several times during this period, and 8.8% once. Among the analyzed socio-demographic and health variables, most of them differentiated responses regarding influenza vaccination in a statistically significant way. In particular: gender (*p* < 0.001), age group (*p* < 0.001); size of place of residence (*p* < 0.001), employment (*p* < 0.001), financial situation (*p* < 0.001), self-assessed health status compared their peer group (*p* < 0.001), and the number of chronic diseases diagnosed by a doctor (*p* < 0.001). However, there was no impact of education (*p* = 0.292), presence of a child in the household: up to 4 years of age (*p* = 0.188); Ages 5–8 years (*p* = 0.656) and 9–12 years (*p* = 0.814) on the seasonal influenza vaccination (Figure 1).

The highest percentage of people declaring influenza vaccination was observed in the 18–24 age group—32.2% (8.5% several times, 23.7% once in the last 3 years). In the remaining age groups, this percentage ranged from 14.7% to 16.1%. A relatively high level of declared influenza vaccination coverage was also observed in the group of people experiencing frequent infections (5 times a year or more)—26.9% (11.5% several times, and 15.4% once). Individuals reporting three or more chronic diseases were also more likely to receive the influenza vaccine—24.3% (13.3% several times and 10.9% once). The lowest vaccination rates were observed among people who believed their health was the same as their peers: 12.7% (6.5% several times and 6.2% once), women: 13.5% (6.2% several times and 7.3% once), and those with the most difficult financial situation: 13.6% (6.4% several times and 7.1% once). Detailed data are presented in Figure 1.

**Figure 2:**
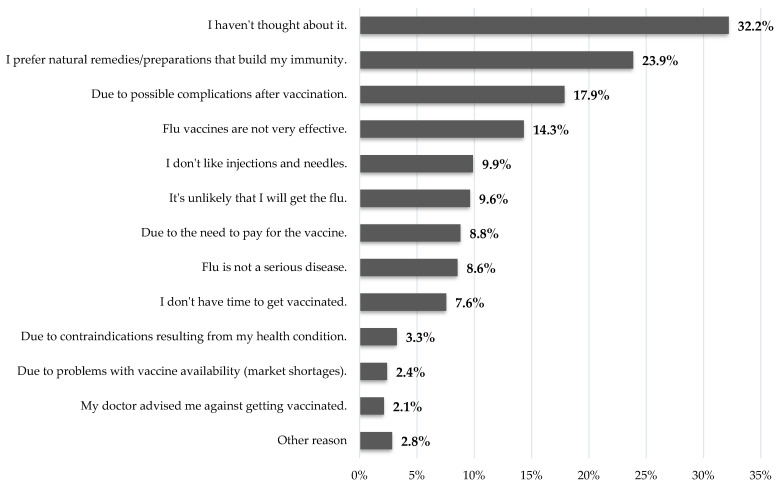
Declarations regarding the reason for not being vaccinated against influenza (in the last 3 years) (only unvaccinated persons, *n* = 4157).

Of all respondents, 83.1% (*n* = 4157) declared that they had not received an influenza vaccination in the last three years. Of this group, 32.2% indicated that they had not considered it at all. Natural remedies/preparations to build immunity were mentioned by 23.9% of this group. Subsequently, possible side effects (17.9%) and the low effectiveness of flu vaccinations themselves (14.3%) were listed. Detailed data are presented in Figure 2.

Analysis of respondents’ opinions regarding statements related to prevention and health showed that these attitudes differentiate responses regarding influenza vaccination in the last three years (Table 2). For all six statements analyzed in this study, the differences were statistically significant (*p* < 0.001). Among those who strongly agreed with the statement that “Vaccinations are unnecessary if someone leads a healthy lifestyle and eats well,” 18.3% had been vaccinated against influenza (9.6% several times, and 8.7% once). Among those declaring they tended to agree, the percentage was 14.6% (7.2% and 7.4%). Among those who strongly disagreed, the percentage was 27.4% (14.0% and 13.4%). Among those who strongly agreed with the statement that “The vast majority of doctors are competent,” the percentage declaring they had been vaccinated against influenza was 28.1% (17.5% and 10.6%). Among those who strongly disagreed with this statement, the percentage was 14.3% (4.0% and 10.2%). Detailed data on the responses for all six items are presented in Table 2. For all six items, the lowest percentage of those declaring vaccination was observed among those who avoided the answer by selecting the “hard to say” option. Depending on the item, those declaring they had been vaccinated against influenza ranged from 7.1% to 12.9%. The percentage of those who selected the “hard to say” answer ranged from 16.3% to 31.5%, depending on the item analyzed (Table 2).

Analysis of reports of influenza vaccination (in the last 3 years) and supplement use in the last 3 months revealed a statistically significant relationship (*p* < 0.001). Among those who used supplements, the percentage of those vaccinated against influenza was 21.8% (11.2% several times, 10.5% once). Among those who used supplements occasionally, the percentage was 14.8% (6.5% and 8.4%). The lowest percentage of those vaccinated was among those who did not use supplements: 12.8% (5.8% and 7.0%) (Table 2).

### 3.2. Factors Associated with Seasonal Influenza Vaccination in the Last 3 Years

The multivariable logistic regression model predicting at least one influenza vaccination in the last 3 years achieved a Cox-Snell R-square of 0.081 and a Nagelkerke R-square of 0.135. Men had 83% higher odds of being vaccinated than women (OR = 1.83; 95% CI: 1.55–2.15). People aged 18–24 had 163% higher odds of being vaccinated than people aged 45–64 (OR = 2.63; 95% CI: 2.05–3.39). Residents of the largest cities (over 500,000 inhabitants) had 44% higher odds of being vaccinated than residents of rural areas (OR = 1.44; 95% CI: 1.13–1.83). People working part-time or on a casual basis had a 37% higher chance of being vaccinated compared to those who did not work (OR = 1.37; 95% CI: 1.08–1.73). For those working full-time, there was no difference compared to those who did not work. The most financially well-off were 64% more likely to be vaccinated than the poorest (OR = 1.64; 95% CI: 1.19–2.24). People who had infections at least five times a year were 92% more likely to be vaccinated than those who reported never getting sick (OR = 1.92; 95% CI: 1.31–2.81). People diagnosed with three or more chronic diseases were 97% more likely to be vaccinated than those without a diagnosed chronic disease (OR = 1.97; 95% CI 1.53–2.53). People who regularly took supplements in the last 3 months were 66% more likely to be vaccinated against influenza compared to those who did not take supplements (OR = 1.66; 95% CI 1.36–2.03). People who believed that most doctors were competent were 206% more likely to be vaccinated than those who avoided clear declarations, choosing the “hard to say” answer (OR = 3.06; 95% CI: 2.27–4.13). Detailed data are presented in Table 3.

## 4. Discussion

This is the first representative study on influenza vaccination coverage rate and public attitudes towards influenza vaccine uptake, which was carried out among working-age adults in Poland. Findings from this study revealed that in December 2024, 16.9% of Poles aged 18–64 years declared getting vaccinated against influenza in the last 3 years, wherein 8.2% were vaccinated several times during this period, and 8.8% were vaccinated once. Male gender, age 18–24 years, living in cities over 100,000 residents, having part-time job, very good household financial situation, frequent infections throughout the year, having chronic diseases, taking dietary supplements regularly and personal beliefs on doctors’ competencies were significantly (*p* < 0.05) associated with getting vaccinated against influenza in the last 3 years.

Poland is recognized as an EU country with one of the lowest influenza vaccination coverage rates [13]. Since 2022, pharmacists have been listed in the law as a group of healthcare professionals who can provide influenza vaccination in community pharmacies [19]. This action was introduced to reduce barriers to access to influenza vaccination and make it possible to acquire the vaccine in pharmacies and arrange vaccine uptake in the same place. In 2024, 40.2% of adults in Poland indicated that if there were such an opportunity, they would be willing to get vaccinated against influenza in the community pharmacy [19]. However, real-world data on influenza vaccination uptake in Poland regularly shows that the percentage of people who get vaccinated is several times lower than those who declared positive attitudes towards vaccination and willingness to get vaccinated against influenza.

This study analyzed influenza vaccine uptake among working-age adults. Findings from this study revealed that 16.9% of Poles aged 18–64 years declared getting vaccinated against influenza in the last 3 years, wherein 8.2% were vaccinated several times during this period, and 8.8% were vaccinated once. Respondents were asked about the influenza vaccine uptake in the last 3 years (December 2022 to December 2024), so the last years of the COVID-19 pandemic were also included in this timeframe. Findings from this study showed that influenza vaccine uptake in working adults is relatively low, and the percentage of people who are vaccinated against influenza regularly (every influenza season) is low (8.2%). Influenza vaccination uptake in working-age adults is similar to that reported in older adults in Poland in 2021 (10%) [13] and slightly higher than in the general population of adults in 2020 (5.5%) [14]. However, influenza vaccination rates among working-age adults are several times lower than rates for the general population (>70%) in Denmark, Ireland, or the Netherlands [13]. Vaccination coverage rates in high-risk groups and healthcare workers in Poland remains low and is below the recommended levels by the public health institutions [13,14].

Lack of interest in influenza vaccination was the major reason for not getting vaccinated. However, 17.9% of those who were not vaccinated reported concerns about the potential side effects of the seasonal influenza vaccine. Also, 14.3% of respondents declared that the influenza vaccine is not effective. The abovementioned observations indicate the presence of misconceptions among the public and a certain degree of misinformation about influenza vaccinations, their validity, and effectiveness [20]. Public health actions are needed to strengthen public knowledge on influenza vaccination and promote evidence-based knowledge on influenza, its prevention methods, and potential complications of the disease.

In this study, multivariable logistic regression analysis revealed 9 different factors associated with getting vaccinated against influenza in the last 3 years. Among those of working age, males were more likely to get vaccinated against influenza. This observation is contrary to previously published data. Samel-Kowalik et al. in a representative sample of adults aged 18 years reported no gender differences in the influenza vaccination coverage rates [14]. Giezeman-Smits et al., who analyzed disparities in seasonal influenza vaccination in Europe, reported higher influenza vaccination coverage rates in females [13]. This observation requires further investigation.

In this study, the youngest age group (18–24 years) presented the highest vaccination coverage rates, especially when asked about the vaccination once in the last 3 years. This may result from the fact that influenza vaccines were offered as an additional vaccine for those who reported for the COVID-19 vaccination within the National Vaccination Program [14]. This observation indicated a high potential to promote influenza vaccines among young adults.

Living in cities with over 100,000 residents and a very good household financial situation was associated with getting vaccinated against seasonal influenza. Their observations are in line with expectations that people with higher economic status and from larger cities are more likely to get vaccinated [21]. Moreover, high-risk groups like those having chronic diseases and those who reported frequent infections throughout the year were more likely to get vaccinated against influenza in the last 3 years. This observation may result from the fact that high-risk groups for influenza complications are often encouraged by healthcare workers to get vaccinated [5]. There were no significant differences in attitudes towards influenza vaccination among those who were working full-time and those who were unemployed. This observation requires further investigation. There was no impact of educational level on attitudes towards getting vaccinated. This may underline the lack of educational gaps in the public knowledge of influenza. Moreover, there was no impact of having children on attitudes towards getting vaccinated against influenza. Small children can play the role of vectors in influenza transmission, but in this study, parents were not more likely to get the influenza vaccine [22].

Those who declared dietary supplement intake were more likely to get vaccinated against influenza. This may result from the higher interest in their own health among those who are taking dietary supplements [23]. Also, public perception of doctors’ competencies was significantly associated with getting vaccinated against influenza. Those who declared trust in doctors’ competencies were more likely to declare getting vaccinated in the last 3 years. This observation underlines the role of physicians in the promotion of seasonal influenza vaccination.

The evolution of some epidemics, such as influenza, demonstrates common patterns both in different regions and from year to year. Rzymski et al. pointed out the need to prioritize vaccinating the unvaccinated individuals and decreasing vaccine inequities rather than optimizing immunity levels in wealthy nations [24]. Boullosa et al. underlined the increased need of new modeling approaches to predict the spread of COVID-19 and influenza in our current world [25]. There is also a need to identify sociodemographic groups that are at lower risk to uptake the influenza vaccine. Data from Saudi Arabia suggest that socio-cultural context may also play a role in decision making on influenza vaccine uptake [26]. Also, attention should be paid to high-risk groups, like those with the risk of atherosclerotic cardiovascular disease, as influenza may lead to serious complications in this population of working adults [27].

This study has several practical implications for vaccination policy. This study revealed the need to strengthen the role of employers and workforce health interventions aimed at seasonal influenza vaccination. Moreover, this study revealed that almost half of those who had been vaccinated in the last 3 years did it once, and only 8.2% of working-age adults were vaccinated for at least two influenza seasons. This observation points out the need to develop public health strategies that encourage those who got vaccinated once to take up the influenza vaccine in the next influenza season. Moreover, this study revealed that socio-demographic and health-related factors are significantly associated with public attitudes towards the influenza vaccine uptake. Targeted educational campaigns are needed to strengthen health literacy levels and improve public knowledge on influenza among those who are less likely to get vaccinated against seasonal influenza. The Global Vaccine Action Plan should better address the needs of low- and middle-income countries as well as in terms of a socio-cultural context [28]. Moreover, potential organizational barriers in influenza vaccination should be identify and removed, especially when related to working adults.

This is a retrospective analysis of a dataset generated in a representative cross-sectional survey. The scope of analysis is limited to records available in the datasets managed by the institution that shared data with the authors. Data on influenza vaccination uptake were self-declared, and medical records were not verified. Respondents were not asked about influenza vaccination campaigns and mobile vaccination points organized in the workplace. Moreover, this study is limited to working-age adults, but data on unemployed people (24.8%) were also included in this study. Lack of a robust correlation analysis between participants’ self-reported vaccination status and their health outcomes, particularly regarding influenza infection is a limitation of this study

## 5. Conclusions

This study revealed very low influenza vaccination coverage rates in working-age adults in Poland. In a group of working-age adults, 9 different factors significantly associated with getting vaccinated against influenza in the last 3 years were identified. The decision to vaccinate against influenza depends on many factors, which should be taken into account when planning educational and information activities. Public health interventions and workforce health promotion programs are needed to address gaps in influenza vaccination uptake among working-age adults. High influenza vaccination coverage rates among working-age adults are necessary to reduce the burden of influenza in Poland.

## Figures and Tables

**Table 1:** Characteristics of the study population (*n* = 5006).

	*n*	%
Overall	5006	100.0
Gender		
male	2506	50.1
female	2500	49.9
Age [years]		
18–24	541	10.8
25–34	990	19.8
35–44	1326	26.5
45–64	2149	42.9
Educational level		
primary or vocational	1593	31.8
secondary	1896	37.9
higher	1518	30.3
Place of residence		
rural area	2035	40.6
city below 100,000 residents	1581	31.6
city 100,000–499,000 residents	789	15.8
city ≥ 500,000 residents	601	12.0
Having children under 4 years of age		
no	4416	88.2
yes	590	11.8
Having children aged 5–8 years		
no	4330	86.5
yes	676	13.5
Having children aged 9–12 years		
no	4342	86.7
yes	664	13.3
Having job (currently working)		
yes, full-time job	2862	57.2
yes, part-time job	902	18.0
no	1242	24.8
Self-assessment of household financial situation		
we have enough money for everything and we are still saving for the future	999	19.9
we have enough money for everything without any special sacrifices but we do not save for the future	977	19.5
we live frugally and thanks to that we have enough money for everything	1805	36.0
we live very frugally in order to save money for more serious purchases	679	13.6
we only have enough money for basic needs or there is not enough money even for the cheapest food	547	10.9
Self-reported health status compared to peers		
definitely better or slightly better	1343	26.8
the same or hard to say	2323	46.4
a little worse	1003	20.0
definitely worse	337	6.7
How often do you get sick/cold?		
not at all (never sick)	460	9.2
1–2 times a year	3125	62.4
3–4 times a year	1057	21.1
5 times a year or more	364	7.3
Number of chronic diseases diagnosed by a doctor		
No disease	2359	47.1
1 disease	1281	25.6
2 diseases	699	14.0
3 or more	667	13.3

**Table 2:** Percentage of people vaccinated against influenza in relation to views on issues related to prevention and health.

Have You Had a Seasonal Influenza Vaccination in the Last 3 Years?					
	*n*	Yes, Several Times	Yes, Once	No	*p*
Dietary supplements are safe and effective for health					
I strongly agree	503	14.9%	13.3%	71.8%	<0.001
I tend to agree	1945	7.3%	8.6%	84.1%	
I tend to disagree	868	8.2%	10.8%	81.0%	
I strongly disagree	297	10.1%	9.4%	80.5%	
It’s hard to say	1393	6.5%	6.1%	87.4%	
Vaccinations are unnecessary if someone leads a healthy lifestyle and eats well.					
I strongly agree	426	9.6%	8.7%	81.7%	<0.001
I tend to agree	869	7.2%	7.4%	85.4%	
I tend to disagree	1391	6.1%	9.3%	84.5%	
I strongly disagree	1294	14.0%	13.4%	72.6%	
It’s hard to say	1025	3.7%	3.4%	92.9%	
Natural therapies, such as herbal medicine, are more effective than synthetic drugs.					
I strongly agree	402	10.0%	8.5%	81.6%	<0.001
I tend to agree	1017	7.3%	8.8%	83.9%	
I tend to disagree	1359	9.0%	9.6%	81.5%	
I strongly disagree	649	10.9%	12.9%	76.1%	
It’s hard to say	1577	6.4%	6.5%	87.1%	
Taking vitamin C in large quantities protects against all types of viral infections					
I strongly agree	481	10.0%	8.5%	81.6%	<0.001
I tend to agree	1416	7.3%	8.8%	83.9%	
I tend to disagree	1382	9.0%	9.6%	81.5%	
I strongly disagree	640	10.9%	12.9%	76.1%	
It’s hard to say	1087	6.4%	6.5%	87.1%	
The vast majority of doctors are competent					
I strongly agree	663	17.5%	10.6%	71.9%	<0.001
I tend to agree	2314	8.1%	9.3%	82.6%	
I tend to disagree	864	6.4%	9.0%	84.6%	
I strongly disagree	322	4.0%	10.2%	85.7%	
It’s hard to say	841	4.3%	5.2%	90.5%	
Once I have a diagnosis, I usually try to get a second opinion from another doctor.					
I strongly agree	416	12.5%	14.2%	73.3%	<0.001
I tend to agree	1322	9.2%	9.5%	81.3%	
I tend to disagree	1818	7.9%	8.6%	83.4%	
I strongly disagree	634	7.1%	10.6%	82.3%	
It’s hard to say	816	5.6%	4.0%	90.3%	

**Table 3:** Multivariable logistic regression model predicting having been vaccinated against influenza at least once in the last 3 years (based on self-reports) (*n* = 5006).

Having Been Vaccinated Against Influenza at Least Once in the Last 3 Years—Yes		
	*p*	OR (95% CI)
Gender		
male	**<0.001**	1.83 (1.55–2.15)
female	Reference	Reference
Age [years]		
18–24	**<0.001**	2.63 (2.05–3.39)
25–34	0.259	1.14 (0.91–1.43)
35–44	0.719	1.04 (0.85–1.28)
45–64	Reference	Reference
Place of residence		
rural area	Reference	Reference
city below 100,000 residents	0.105	0.85 (0.7–1.03)
city 100,000–499,000 residents	**<0.05**	1.3 (1.04–1.62)
city ≥ 500,000 residents	**<0.01**	1.44 (1.13–1.83)
Having job (currently working)		
yes, full-time job	0.913	0.99 (0.8–1.22)
yes, part-time job	**<0.01**	1.37 (1.08–1.73)
no	Reference	Reference
Self-assessment of household financial situation		
we have enough money for everything and we are still saving for the future	**<0.01**	1.64 (1.19–2.24)
we have enough money for everything without any special sacrifices but we do not save for the future	0.098	1.31 (0.95–1.8)
we live frugally and thanks to that we have enough money for everything	0.292	1.17 (0.87–1.58)
we live very frugally in order to save money for more serious purchases	0.275	1.21 (0.86–1.69)
we only have enough money for basic needs or there is not enough money even for the cheapest food	Reference	Reference
Self-reported health status compared to peers		
definitely better or slightly better	0.116	1.31 (0.93–1.85)
the same or hard to say	0.092	0.75 (0.54–1.05)
a little worse	0.762	0.95 (0.68–1.33)
definitely worse	Reference	Reference
How often do you get sick/cold?		
not at all (never sick)	Reference	Reference
1–2 times a year	0.917	1.02 (0.76–1.36)
3–4 times a year	**<0.05**	1.42 (1.02–1.96)
5 times a year or more	**<0.01**	1.92 (1.31–2.81)
Number of chronic diseases diagnosed by a doctor		
No disease	Reference	Reference
1 disease	**<0.01**	1.32 (1.08–1.61)
2 diseases	**<0.05**	1.39 (1.08–1.77)
3 or more	**<0.001**	1.97 (1.53–2.53)
Taking dietary supplements in the last 3 months		
yes, regularly	**<0.001**	1.66 (1.36–2.03)
yes, occasionally	0.702	1.04 (0.84–1.3)
no	Reference	Reference
Most doctors are competent		
I strongly agree	**<0.001**	3.06 (2.27–4.13)
I tend to agree	**<0.001**	1.77 (1.36–2.3)
I tend to disagree	**<0.05**	1.41 (1.04–1.91)
I strongly disagree	0.352	1.21 (0.81–1.82)
It’s hard to say	Reference	Reference

## Data Availability

This study is a secondary data analysis. Dataset available on request from the authors.

## Supplementary Materials

The following supporting information can be downloaded at: https://www.mdpi.com/article/10.3390/vaccines13090954/s1, File S1: List of questions used for the purposes of this study.
