# Supplementary material for: Factors Associated with Seasonal Influenza Vaccination Among Working-Age Adults in Poland: A Nationwide Cross-Sectional Survey
Source: Vaccines (Basel). 2025 Sep 6;13(9):954. doi: 10.3390/vaccines13090954 (PMC12474479; doi:10.3390/vaccines13090954)
Supplement: Supplementary file 1 [file vaccines-13-00954-s001.zip › Supplementary File S1.pdf]

P1. Respondent's gender:

a) Male
b) Female

P2. In what year were you born? _ _ _ _

P3. What is your education level? Please provide the highest level of education you have achieved.

a) rural area
b) city below 100,000 residents
c) city 100,000-499,000 residents
d) city >=500,000 residents

P4. What is your education level? Please provide the highest level of education you have achieved.
e) Unfinished primary or no school education
f) Primary
g) Lower secondary
h) Basic vocational (also SPR)
i) General secondary without matriculation exam
j) General secondary with matriculation exam
k) Vocational secondary without matriculation exam
l) Vocational secondary with matriculation exam (technical school, vocational or technical high school)
m) Post-secondary or post-secondary
n) Higher education with the title of engineer, bachelor, certified economist
o) Higher education with a master's degree, medical degree or equivalent
p) Higher education with a doctoral degree or higher

P5. Are you currently gainfully employed (full-time, in your own company or farm or do you perform commissioned work)?
a) Yes, full-time
b) Yes, part-time
c) Yes, occasional
d) No

P6. Which of the following best describes the financial situation of your household?
a) We have enough for everything and we are also saving for the future
b) We have enough for everything without any special sacrifices but we are not saving for the future
c) We live frugally and thanks to this we have enough for everything
d) We live very frugally to save for more serious purchases
e) We only have enough money for basic needs
f) We do not have enough money even for the cheapest food

P7. Do you suffer from any of the following diseases? [YES/NO/I DON'T KNOW - for each]
a) Allergies (e.g. skin allergies, hay fever)
b) Food intolerance, food allergy
c) Urinary tract diseases (urolithiasis, kidney failure, glomerulonephritis, other kidney or urinary tract diseases)
d) Circulatory system diseases (hypertension, previous stroke, previous myocardial infarction, coronary artery disease, heart failure, atherosclerosis of the lower limb arteries, lipid disorders (high: total cholesterol, LDL, triglycerides), other heart diseases)

e) Musculoskeletal diseases (joint disease, osteoporosis, other)
f) Type 1 diabetes
g) Type 2 diabetes
h) Digestive system diseases (peptic ulcer disease, gastroesophageal reflux disease, bowel disease, liver disease, pancreas disease, gallstone disease biliary, other)
i) Respiratory diseases (COPD, tuberculosis, asthma, other)
j) Endocrine diseases (hypothyroidism, hyperthyroidism, other)
k) Neurological diseases (Parkinson's disease, multiple sclerosis, other)
l) Cancer
m) Skin diseases (including psoriasis)
n) None of the above

P8. On average, how often during the year are you sick or have a cold, excluding situations resulting from a chronic disease)
a) I never get sick
b) 1-2 times a year
c) 3-4 times a year
d) 5-6 times a year
e) 7-12 times a year
f) More than 12 times a year

P9. Is my health in comparison to people my age?
a) Definitely better
b) A little better
c) The same
d) A little worse
e) Definitely worse
f) Hard to say

P10. In the past three years, have you received an influenza (flu) vaccination?
a) Yes, more than once [SKIP TO NEXT SECTION]
b) Yes, once [SKIP TO NEXT SECTION]
c) No

P11. What were your main reasons for not receiving an influenza vaccination? Please select no more than three of the most important reasons.
[MULTIPLE RESPONSE ALLOWED]
a) I believe I am unlikely to contract influenza
b) I do not consider influenza to be a serious illness
c) I believe influenza vaccines are not very effective
d) I am concerned about potential adverse effects of the vaccine
e) I have medical contraindications to vaccination
f) I do not have time to get vaccinated
g) I have a fear of injections or needles
h) I did not want to pay for the vaccine
i) The vaccine was not available (e.g., market shortages)
j) I prefer natural remedies or supplements to support my immune system
k) My doctor advised against vaccination
l) I have not given it much thought
m) Other reason (please specify)

P12. In the past three months, have you taken any vitamins, dietary supplements, minerals, or products containing herbal ingredients?
p) No
q) Yes, regularly
r) Yes, occasionally

P13. Please indicate whether you agree or disagree with the following statements:

1 - I strongly agree; 2 - I tend to agree; 3 - I tend to disagree; 4 - I strongly disagree; 5 - It's hard to say

(1) Dietary supplements are safe and effective for health

(2) Vaccinations are unnecessary if someone leads a healthy lifestyle and eats well

(3) Natural therapies, such as herbal medicine, are more effective than synthetic drugs

(4) Taking vitamin C in large amounts protects against all viral infections

(5) The vast majority of doctors are competent

(6) After receiving a diagnosis, I usually try to get a second opinion from another doctor
